# Continuously Grooved Stent Struts for Enhanced Endothelial Cell Seeding

**DOI:** 10.1007/s00270-017-1659-4

**Published:** 2017-05-03

**Authors:** Marja ter Meer, Willeke F. Daamen, Yvonne L. Hoogeveen, Gijs J. F. van Son, Jeremy E. Schaffer, J. Adam van der Vliet, Leo J. Schultze Kool, Lambertus P. van den Heuvel

**Affiliations:** 1 Department of Radiology and Nuclear Medicine (766), Radboud University Medical Center, PO Box 9101, 6500 HB Nijmegen, The Netherlands; 2 Department of Biochemistry 280, Radboud Institute for Molecular Life Sciences, Radboud University Medical Center, PO Box 9101, 6500 HB Nijmegen, The Netherlands; 3 Fort Wayne Metals, Research and Development, 9609 Ardmore Avenue, Fort Wayne, IN 46809 USA; 4 Department of Surgery 618, Radboud University Medical Center, PO Box 9101, 6500 HB Nijmegen, The Netherlands; 5 Department of Pediatrics/Pediatric Nephrology 774, Radboud University Medical Center, PO Box 9101, 6500 HB Nijmegen, The Netherlands; 6 Department of Development and Regeneration/Pediatrics, Catholic University Leuven, PO Box 7003, 3000 Leuven, Belgium

**Keywords:** Endovascular stent, Endothelialization, 316L stainless steel, Nitinol, Cell adhesion, In vitro

## Abstract

**Purpose:**

Implantation of pre-endothelialized stents could enhance cellular recovery of a damaged vessel wall provided attached cells remain viable, functional and are present in sufficient numbers after deployment. The purpose of this study was to evaluate the feasibility of grooved stainless steel (SS) stents as a primary endothelial cell (EC) carrier with potentially enhanced EC protection upon stent deployment.

**Materials and Methods:**

Attachment and behavior of enzymatically harvested human adult venous ECs seeded onto gelatin-coated vascular stents were evaluated in an in vitro setting. Smooth and grooved SS stents and smooth nitinol stents were studied.

**Results:**

All cells expressed EC markers vWF and CD31. Using rotational seeding for a period of 16–24 h, ECs attached firmly to the stents with sufficient coverage to form a confluent EC monolayer. The grooved SS wire design was found to enable attachment of three times the number of cells compared to smooth wires. This also resulted in an increased number of cells remaining on the stent after deployment and after pulsatile flow of 180 ml/min for 24 h, which did not result in additional EC detachment.

**Conclusions:**

The grooved stent provides a potential percutaneous means to deliver sufficient numbers of viable and functional cells to a vessel segment during vascular intervention. The grooves were found to offer a favorable surface for EC attachment and protection during stent deployment in an in vitro setting.

**Electronic supplementary material:**

The online version of this article (doi:10.1007/s00270-017-1659-4) contains supplementary material, which is available to authorized users.

## Introduction

Despite the fact that vascular interventions offer a life- and limb-saving treatment for millions of patients each year, treated vessels are still prone to re-occlusion. The devices inserted during these procedures inadvertently cause cellular damage to the endothelial cell (EC) lining of the vessel wall and the tunica media, triggering a cascade of events that contribute to restenosis [1]. These events include platelet activation, thrombus formation and smooth muscle cell (SMC) proliferation. After complete re-endothelialization, these reactive events subside [2–4].

Natural restoration of the EC layer is slow, particularly in humans [5]. When artificial biomaterials are applied (e.g., stents or grafts), healing is impaired even further [6]. Although initial technical and clinical success rates using current intervention technologies are reported to be high, patency rates after two years for balloon angioplasty and stenting of peripheral femoral arteries are much lower (25–87%) [7]. Late stent thrombosis can impair vessel patency in the long term [1, 8]. Furthermore, although the advent of anti-proliferative drugs has significantly reduced problematic SMC invasion, concerns of late thrombotic events due to insufficient re-endothelialization remain [9].

Biomaterials containing or attracting endothelial cells have been applied in various preclinical and clinical studies in an effort to reduce neointimal proliferation [10]. Examples of cell types used for in vitro studies include human umbilical vein endothelial cells (HUVECs), endothelial progenitor cells and genetically engineered cells [11–15]. Another approach to improve re-endothelialization in vivo entails the use of superficial nano- or microscale surface features to alter surface roughness, either by using surface coatings or by engineering these features onto the metal itself [16, 17]. These surface modifications favor EC migration and thus endothelialization [16]. Porous delivery vehicles for pre-endothelialization of catheter-loaded stents have recently been developed, although cellular damage during balloon deployment remains a limiting concern [18].

In the current in vitro study, for the purpose of rapid in vivo re-endothelialization after endovascular procedures, a novel metal stent with continuous helically grooved struts is compared to conventional smooth surface stents. The aim is to gage suitability as a carrier of autologous ECs in which the cells are protected during deployment in an effort to innovate delivery and state-of-the-art materials technology for real patient benefit in the future. The goal was to develop a method that allows for the use of a patient’s own endothelial cells and minimizes clinical preparation time.

## Materials and Methods

All experiments were performed at least three times in independent experiments (*n* = 3) with cells from at least two human donors. Remnant surgical waste tissue was used for these experiments. Under Dutch law, use of such remnant tissue for research purposes is permitted. As all donor tissue was processed anonymously, donor characteristics are unknown.

**Fig. 1:**
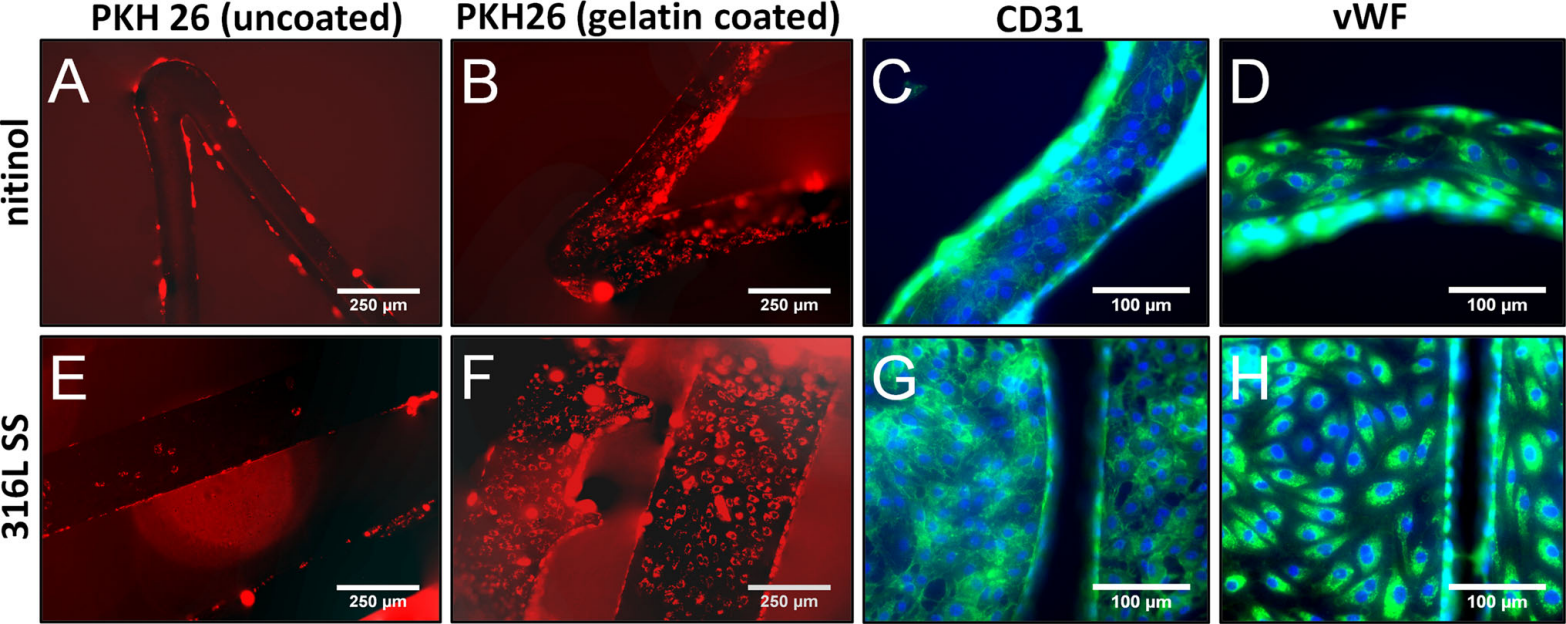
Summary of preliminary experiments: Optimization of surface coating using gelatin for EC seeding onto stents and characterization of attached ECs. Attachment of ECs to gelatin-coated stents, both nitinol and 316L SS, was superior over attachment to uncoated stents after a 24 h seeding period (**A**, **B**, **E**, **F**). Cells were stained with a fluorescent membrane marker PKH26 for visualization. Attached cells had a proper phenotype as EC-specific markers von Willebrand factor and CD31 (PECAM-1) were expressed (both *green*), cells were counterstained with DAPI in *blue* (**C**, **D**, **G**, **F**)

As an integral part of the assessment of the novel grooved surface geometry versus smooth surface geometry, initial experiments were performed to determine suitable conditions for EC attachment and proliferation using various reject or sample stents. Detailed descriptions of these experiments were omitted here for the sake of clarity, and a summary can be found in the results section and in Fig. 1.

### Cell Isolation and Culture

Human vein segments of 2–4 cm, remnant tissue from organ donations (*n* = 14), were used for EC isolation. EC growth medium 2 (EGM-2) with EGM-2 SingleQuots (Lonza, Walkersville, MD, USA) was used. The fetal bovine serum from the kit was replaced with 10% human AB serum (Seralab, Haywards Heath, UK), henceforth referred to as EGM-2+. The veins were stored in phosphate-buffered saline (PBS) (pH 7.4) at 4 °C, cannulated within 6 h after surgery, flushed with 25 °C PBS and filled with 0.6 mg/ml collagenase type 2 (Worthington, Lakewood, NJ, USA) at 37 °C for 30 min. Veins were then flushed with PBS; the cell suspension was collected and washed twice in EGM-2+ at 350 g. Harvested cells were seeded in EGM-2+ onto 10 cm^2^ polystyrene plates coated with 2% (w/v) porcine gelatin (300 bloom, Sigma, St Louis, MO, USA, dissolved in purified water and sterilized using steam autoclaving). Cells were not counted after isolation to limit cell loss. After the first passage, low-density plating was applied (1:10), and medium was refreshed every 2–3 days. All cells were cryopreserved between passage 2 and 6 and stored in liquid nitrogen until further use. All experiments were performed using cells between passages 3 and 8. Cells were kept in culture up to 14 passages to study cell characteristics in higher passages. It should be noted that cell seeding took place under hypoxic conditions and that experiments were performed without an additional culture period. Immunocytochemistry was used to visualize EC phenotype (Fig. 1), details of this experimental procedure can be found in the supplementary files.

### Stents

**Fig. 2:**
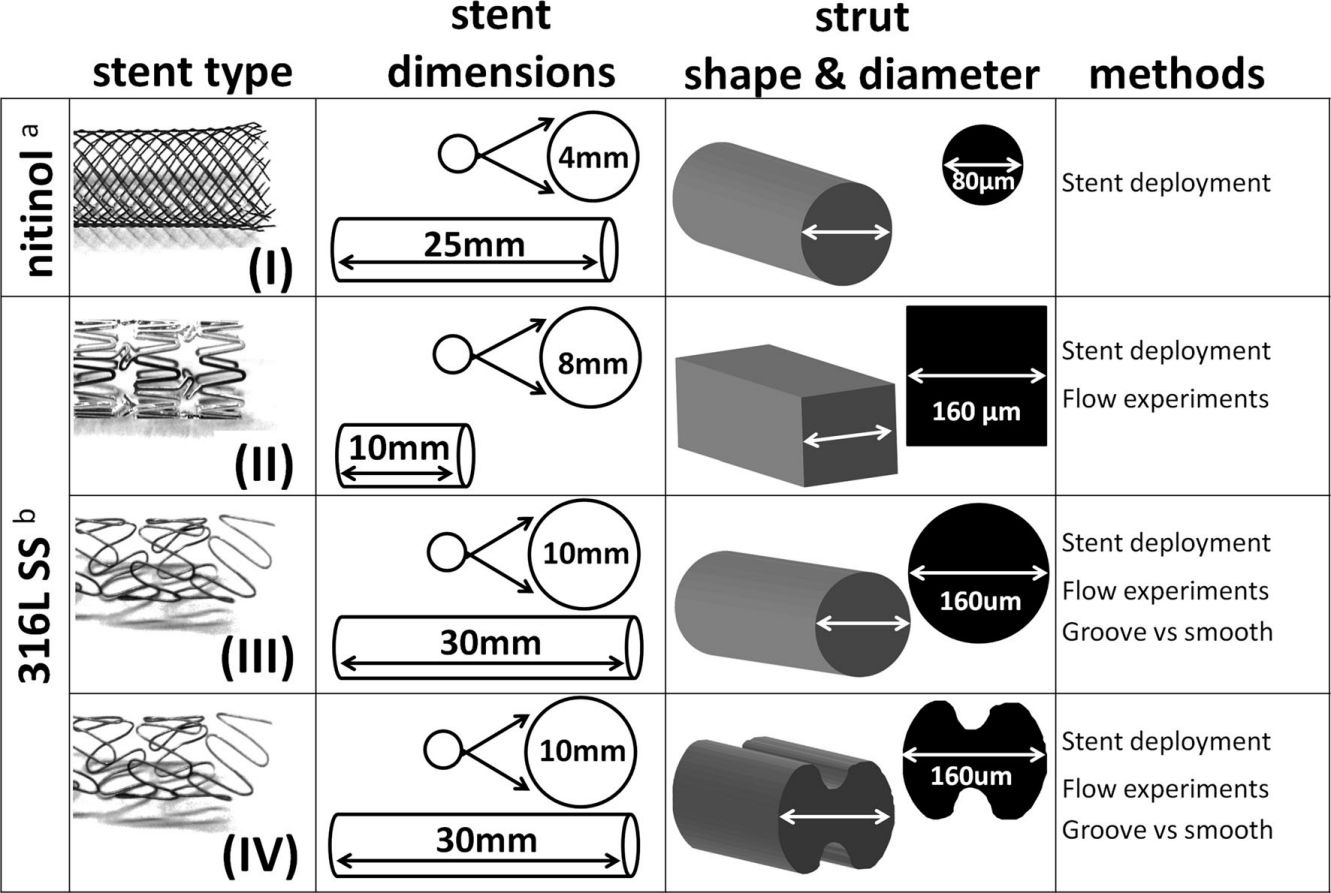
An overview of the stent design and dimensions of stents and struts used in the current study. ^a^Self-expanding, ^b^balloon expandable

Figure 2 depicts all the details regarding stent design and dimensions of the stents. Stents of type I (nitinol), III (316L SS) and IV (316L SS) were custom-built (Fort Wayne Metals, Fort Wayne, Indiana, USA). Stents of type II (316L SS, Constant, Alvimedica, Istanbul, Turkey) were kindly provided by the manufacturer. Custom SS round strut wire stents (stents III and IV) were made with and without ∅ 40 µm grooves along the entire length of the wire for the purpose of analyzing the protection of ECs within the grooves of the stent struts. Custom wires were produced using conventional and bulk-replicable methods in a state-of-the-art medical wire production facility.

**Fig. 3:**
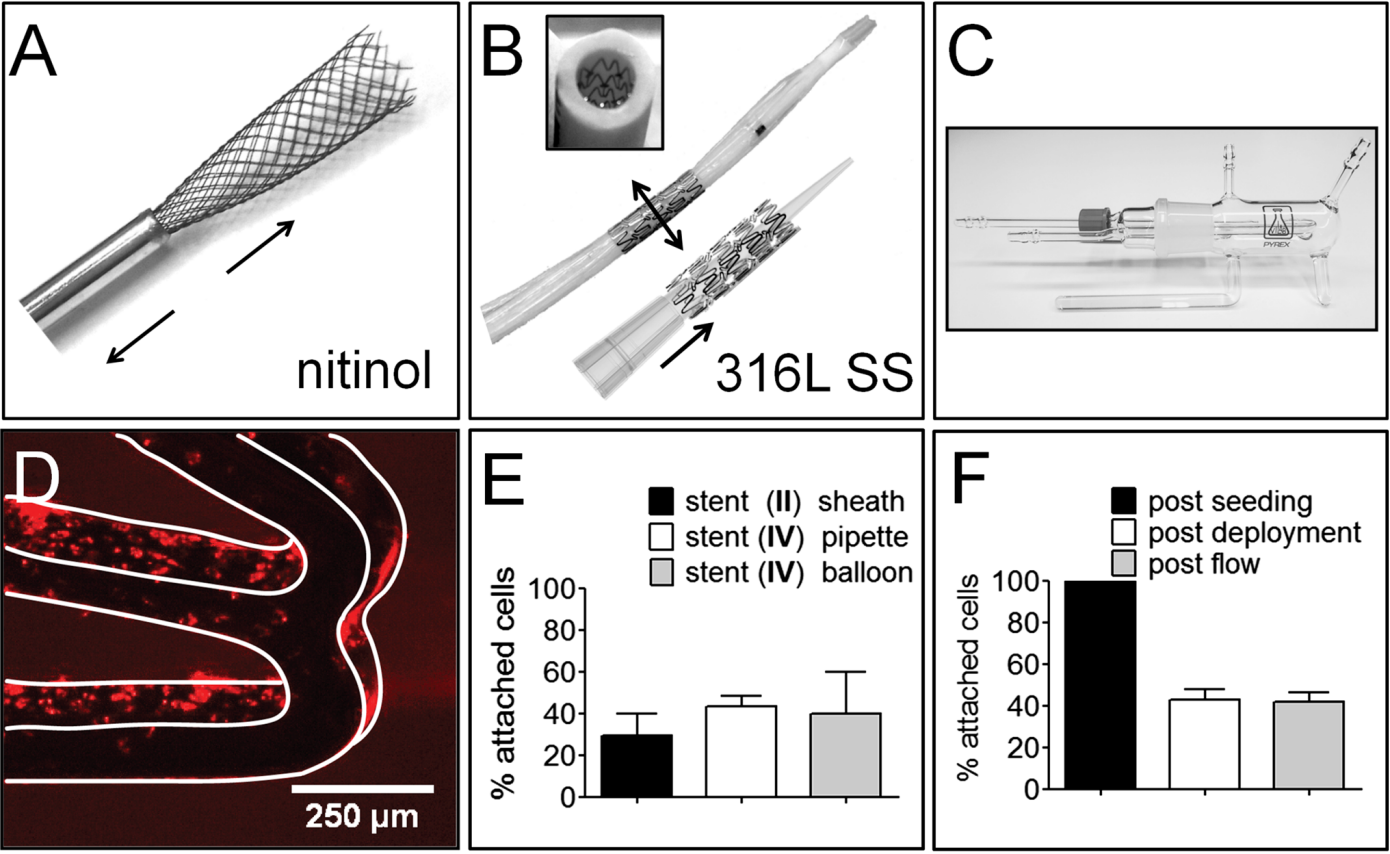
Stent deployment resulted in a loss of seeded EC for all stents, subsequent exposure to flow did not result in additional cell loss. **A** Stent (I) deployment by pulling the stent through a small tube. **B** Stent (II) deployment inside a PharMed tube using either an angioplasty balloon (*top*) or a pipette tip (*bottom*). **C** The tubular glass chamber used for flow experiments. **D** The stent outline is highlighted to demonstrate loss of cells on the lateral side of stent (II). **E** Due to stent deployment, a cell loss of over 50% of the cells was observed based on a CCK-8 assay. **F** The CCK-8 assay confirmed there was minimal additional cell loss from the stents (II) after exposure to flow (2.0 ± 2.2%; *n* = 5)

**Fig. 4:**
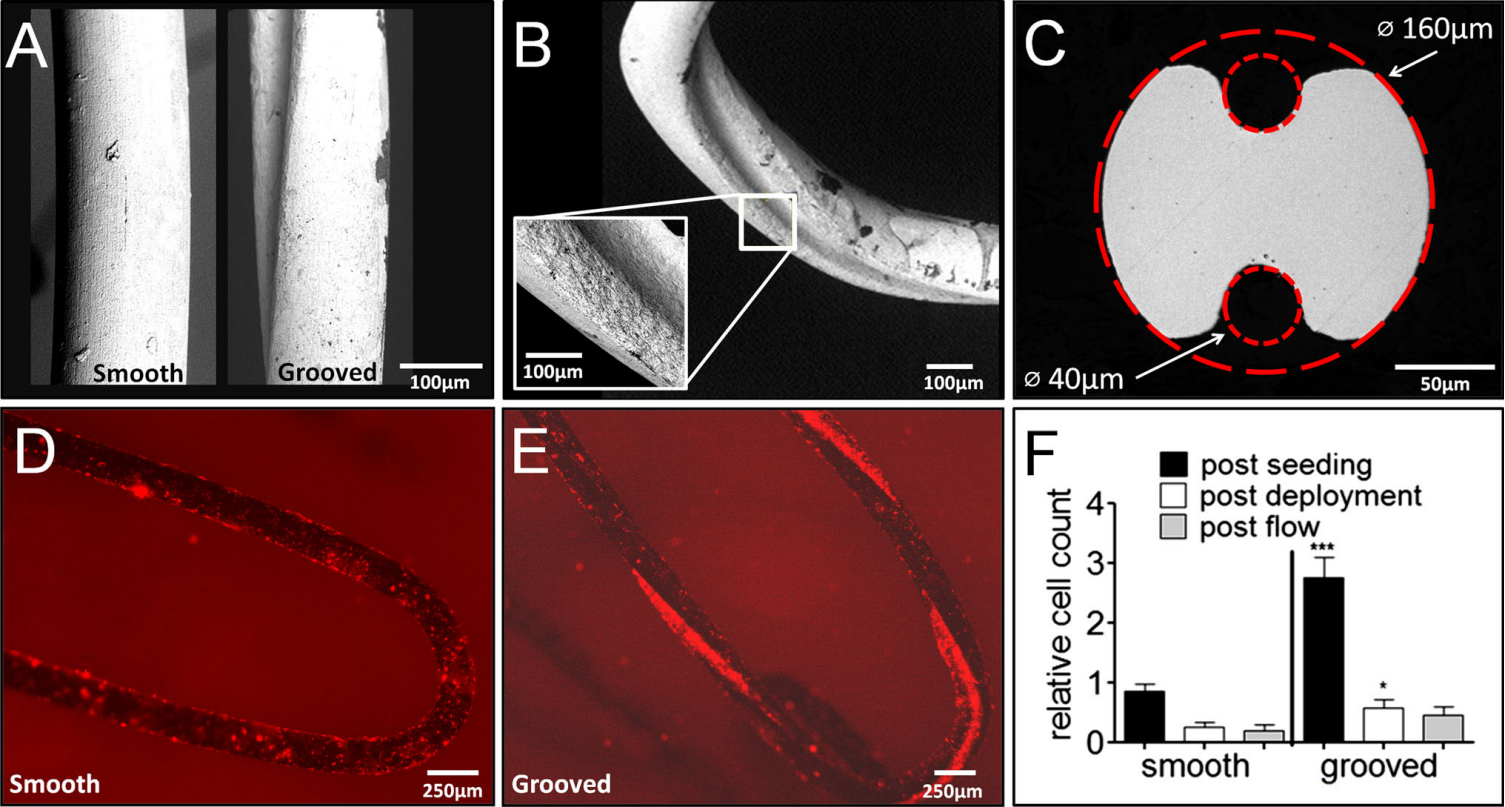
Stents (IV) consisting of 316L wire afforded with grooves can accommodate three times more cells compared to a 316L stent (III) with the same size and the same design but without grooves. **A** SEM images of the smooth and grooved stent struts. **B** SEM image showing the continuous helical groove. **C** Groove design, ∅ 40-µm grooves resulting in an area increase of 20%. **D** Cells were labeled with PKH26, cells covered the entire surface of the smooth stent. **E** A very dense cell population was observed within the grooves compared to the rest of the surface. **F** Based on the CCK-8 assay, the grooved stent could accommodate 3.2 ± 1.4 times as many cells as the smooth stent, and the relative cell count after deployment was also higher for the grooved stent

### Cell Seeding Onto Stents

In order to visually track the attached living cells over time, all cells were labeled with the fluorescent cell marker PKH-26 (Sigma), according to the standard product protocol, prior to seeding onto metal (Fig. 1). Stents were cleaned by sonication (Branson 2200, Danbury, CT, USA) by consecutive immersions in 20% w/v citric acid (Sigma), demineralized water and 99% isopropanol (Merck Millipore, Billerica, MA, USA) for 5 min at 40 kHz [19]. Cleaned stents were sterilized by high-pressure steam autoclaving for 15 min at 121 °C. Sterilized stents were incubated with 2% (w/v) gelatin in demineralized water for 2 h at 37 °C in sealed 2-ml cryovials, which were swirled every 10 min. All stents were washed once with PBS prior to seeding with 2 × 10^6^ cells in 1 ml EGM-2+ in a 2-ml cryotube to completely immerse the stents (6 × 10^6^ cells/cm^2^ metal). The sealed vials were rotated continuously at 10 rpm at 37 °C (16–24 h) to obtain confluent EC coverage.

The cell counting kit (CCK-8, Sigma) is non-toxic to cells, which allowed its consecutive use on a single stent to determine the number of attached cells immediately after seeding, after stent deployment and after exposure to flow. Manually counted cells, using a counting chamber, were used for a calibration curve. 10% (v/v) CCK-8 in EGM-2+ was added to the stents in a 24-well or 96-well plate for 3 h, and absorbance of the medium at 450 nm was measured using the Bio-Rad 550 (Bio-Rad, Hercules, CA, USA) or the Bio Tek ELx800 (Bio Tek, Winooski, VT, USA). Stents were washed in EGM-2+ subsequently used in experiments to mimic stent use in vivo.

### Simulated Stent Use

#### Stent Deployment

Figure 3 illustrates the methods of deployment. The self-expanding woven nitinol stents (I) were stretched to fit inside a metal tube (∅ 1 mm) and pulled through to mimic stent deployment from a self-expansion delivery sheath (Fig. 3A). The SS stents (II) were deployed by insertion of a sterile pipette tip to provide smooth expansion. In vivo use of SS stents requires balloon expansion for deployment in a blood vessel; therefore, these stents and stents III and IV were also hand-crimped onto a ∅ 7-mm balloon that was used to deploy the stents inside a ∅ 4-mm tubular PharMed tube (Saint-Gobain, Courbevoie, France). Scanning electron microscopy images were taken using the Phenom Pure (Phenom-World, Eindhoven, the Netherlands).

#### Resistance to Flow

In an in vivo environment, the cell-seeded stents will be exposed to the bloodstream and should be flow resistant. Blood flow was simulated with EGM-2+ medium in tubular flow chambers (Ebers Medical, Zaragoza, Spain), using a peristaltic pump (two rollers, flow 120–200 ml/min, Mellor Electrics, Blackburn, UK) and power supply Basetech BT-305 (Fig. 3C). SS stent deployment using the pipette tip method was performed in a sterile flow cabinet, using a ø 4-mm PharMed tube as an outside barrier. The wall shear stress can be estimated according to: *τ* = 4*Qη/πr* ^*3*^, where *τ* is wall shear stress in dyne/cm^2^; *Q* is flow rate (180 ml/min or 3 cm^3^/s); *η* is the dynamic viscosity of the medium (0.008 g cm^−1^ s^−1^ [20]); and r is the radius of the stent lumen (0.2 cm) [21]. A shear stress of 0.38 Pa (3.8 dyne/cm^2^) was applied. This estimation assumes steady instead of pulsatile flow.

#### Outgrowth of Seeded Cells

**Fig. 5:**
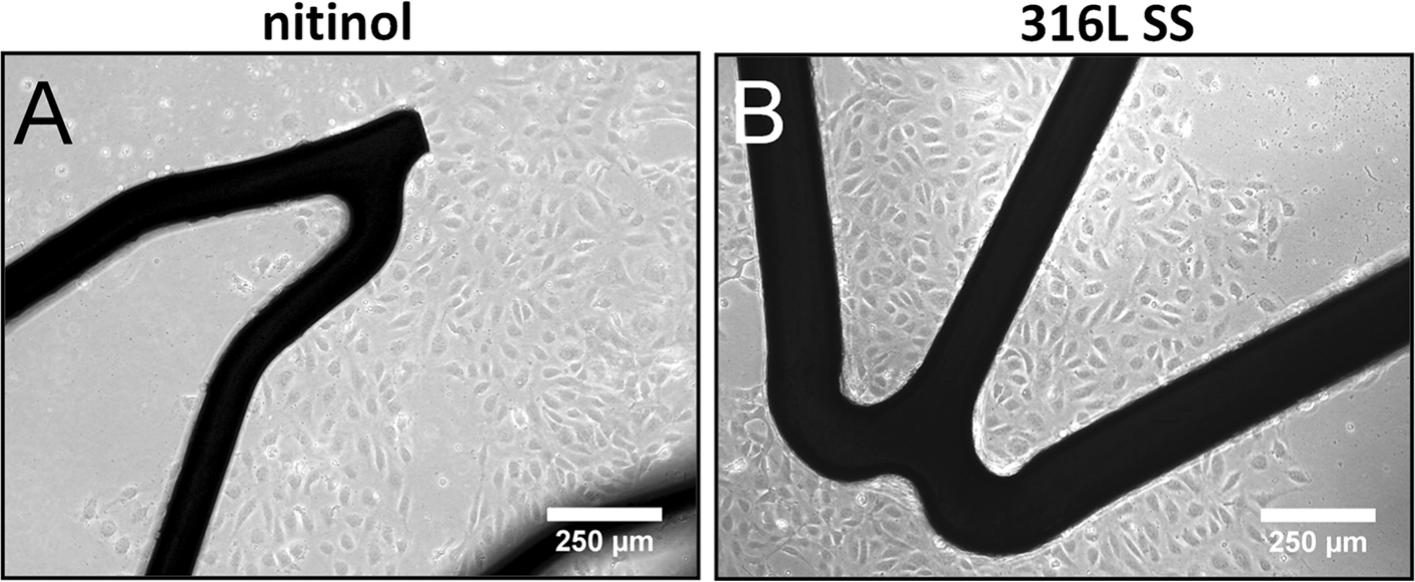
ECs seeded onto gelatin-coated stents (I) and (III) are still proliferative and able to endothelialize a surface beyond the stent surface. **A**, **B** phase contrast images after 48 h in culture

When applying a cell-seeded stent in vivo, the blood vessel inner lumen outside the mesh framework will be denuded. These areas of native tissue without ECs need to be covered by endothelial cells growing out from the stent. To simulate this situation in vitro, cell-seeded stents were placed in a gelatin-coated culture flask to study cell outgrowth. Cell coverage of the gelatin-coated surfaces was qualitatively assessed once a day using a Leica DMIL LED phase contrast microscope for 7 days (Fig. 5).

#### Data Analysis

All quantitative data are expressed as mean ± standard deviation. All experiments were performed at least three times; in case more than three experiments were performed, the specific number is stated in the results. Student’s one-tailed unpaired *t* tests were performed to determine statistical significance using GraphPad Prism software version 5.03 (La Jolla, CA, USA). Results were considered statistically significant with *p* < 0.05, where the significance level is indicated by * for *p* < 0.05, ** for *p* < 0.01 and *** for *p* < 0.001.

## Results

Figure 1 gives an overview of all pilot experiments that gave shape to the experimental design reported here. Application of a coating with gelatin to both the nitinol and SS stents resulted in enhanced cell adhesion compared to bare metal surfaces. The EC-specific staining showed that the isolated cells were of the endothelial phenotype; both Weibel–Palade bodies (vWF) and intercellular junctions (CD31) were clearly present in cells attached to stents.

### EC Isolation and Culture

ECs derived from human veins showed characteristic EC cobblestone morphology. Subculture up to passage 14 was possible without loss of phenotype. On average, 200,000 cells were available after 7 days, 2 million after 12 days and 20 million cells after 17 days. EC proliferation was delayed at subculture densities below 1:10, whereas at densities below 1:25 growth cessation was observed. Cryopreservation did not influence EC proliferation.

### EC Attachment to Stents

After overnight seeding (16–24 h), nitinol stents (I) were completely covered with cells and contained between 2000 and 3500 cells. Stents (II) with a visually confluent EC layer contained 4000–6500 cells per stent based on the CCK-8 assay, which translates to 16,000 ± 2400 cells per cm^2^ (*n* = 9). To reach confluency, at least 2 × 10^6^ cells had to be seeded per stent with a total surface area of 34.08 mm^2^.

The grooved stent was designed based on the observation that cells seeded on the surface of stents were prone to detach when mechanical force was applied. The groove in the wire not only increased the surface area of stent IV by about 20% compared to stent III, but also provided concave attachment surfaces (Fig. 4). After seeding of ECs, the entire stent was covered with cells, including the grooves within the stent wire (Fig. 4B, C). The stent with the grooved wire could accommodate three times more cells than the similarly sized smooth stent (3.2 ± 1.4; *p* < 0.001). The smooth stents (III) contained between 1000 and 2000 cells, the grooved stents (IV) between 2000 and 5000 cells.

### Simulated Stent Use

#### Stent Deployment

In order to assess the attachment of endothelial cells seeded onto the stents after deployment, stents were subjected to simulated stent use. Self-expanding stents (I) that were pulled through a small tube (Fig. 3A) showed cell detachment of 71 ± 18% (Fig. 3E). On these stents, the observed cell loss was more diffuse compared to the SS stents. SS stent (II) deployment using a pipette tip resulted in cell loss of 57 ± 10%, and balloon deployment resulted in cell detachment of 60 ± 19%, results were not statistically significant (Fig. 3B, E; *p* = 0.2). The balloon expandable stents (II) were deployed inside a ∅ 4-mm tubular PharMed tube to mimic deployment inside a blood vessel. This resulted in preservation of cells on the lateral sides of the stent struts, but loss of cells at the external side and the luminal side of the stents where the stents came into contact with the tube and the balloon or pipette tip (Fig. 3D).

Due to hand crimping and balloon deployment, 85 ± 10% of cells were lost from the grooved stent (IV), which is a similar proportional loss as the smooth stents (III) but with overall higher cell retention numbers. The addition of the grooves tripled the final cell count, *p* = 0.05 (Fig. 4F).

#### Resistance to Flow

After deployment, all SS stents were exposed to physiological flow of 3.8 dyn/cm^2^ for 24 h, which resulted in minimal additional cell loss for type II stents (2.0 ± 2.2%; *n* = 5) (Fig. 3F). Additional cell loss due to flow for the smooth stents (III) was comparable to the grooved stents (IV), highlighting that the majority of attached cells remained attached under flow conditions (21 ± 17% and 22 ± 11%; *p* = 0.2).

#### EC Outgrowth from Stents

Cell proliferation and outgrowth from the coated stents was observed within 24 h and continued until the culture flask surface was confluent. Stents that were kept in culture for 2 weeks were still covered with cells, showing that cells indeed grew out from the stent and did not just migrate away from the stent (Fig. 5).

## Discussion

One of the oldest concepts in the field of tissue engineering, endothelial cell seeding, has as yet not succeeded in achieving clinical application in vascular regenerative medicine. Our results suggest that stents manufactured from grooved compared to smooth wire can allow for the implantation of more cells, thus increasing the potential for rapid re-endothelialization after vascular intervention. In order to make this method more suitable for clinical practice, further investigation is being undertaken. Wire shape and groove design will be the main focus toward improved seeding efficiency and to reduce cell loss due to physical contact with other devices during deployment. Other opportunities include the use of absorbable materials and self-expanding grooved designs. When these technical improvements are realized, EC-seeded endovascular stents with a grooved surface geometry could indeed provide a means to induce accelerated vascular repair.

Mature ECs have the reputation of being senescent and difficult to maintain in culture [22, 23]. In our hands, however, these cells were easy to isolate and maintain. Toward unintrusive practice, current endoscopic techniques allow for minimally invasive vein harvesting under only local anesthesia. A limitation of this study is the use of healthy donor cells, which may not reflect results in a real-world patient population. However, Deutsch et al., using a different culture medium, reported only 2.5% growth failure for patient derived cells (*n* = 318). Based on this study the cephalic, basilic or saphenous veins would be suitable options for cell harvesting in future clinical application [24].

Dense cell attachment within concavities has been reported [16, 17, 25]. The purpose of these surface modifications was to speed up endothelialization of the actual stents by ECs still present in the vessel. We are not the first to use cell-seeded stents as a delivery vehicle [11, 12, 15, 21]. However, to our knowledge the current study is the first report of cell attachment to a continuous helical groove feature, with the purpose of using seeded cells to re-endothelialize the denuded vessel. The continuity of the feature along the entire strut length is important in stent design for predictable mechanical behavior including fatigue endurance. Given the threefold increase in cell attachment in comparison with the modest 20% enhancement of strut area, other factors must also be responsible for the increased cell numbers. One mechanism may be the augmented texture within the groove, as ECs are known to prefer a more textured surface [16, 17]. Further, the influence of convex versus concave attachment surfaces is suggested to affect both attachment and functional behavior [26]. Stents are commonly polished to create a smooth surface, as this is believed to decrease thrombogenicity. However, a direct comparison of smooth and rough surface stents in a clinical study showed that rough stent surfaces did not increase late lumen loss after stent implantation [27]. Another potential benefit is that rough or textured surfaces may inhibit smooth muscle cell proliferation while stimulating EC proliferation [28]. More work is needed to understand long-term implications of concavities and texture on cell function. Effective design for topographically driven adherence may well eliminate the need for coatings such as gelatin.

A seeding period of 24 h is considerably shorter than the previously described period of days to weeks required for cells seeded onto vascular grafts to become flow resistant [24]. In our setup, the additional maturation period after the initial 24 h seeding period was eliminated because cells were immediately resistant to flow. However, we did not study the potential beneficial effect of a longer maturation period on the cell detachment due to mechanical interaction. A limitation of this study is the period of flow exposure of only 24 h to culture medium. Future work examining both flow duration and viscosity effects is warranted.

The fact that that a large number of cells detached upon “delivery” is a limitation of the current study. The relatively small number of successfully delivered cells decreases the potential re-endothelialization speed, and further, the detached cells might aggregate and cause problems downstream. However, this problem should be placed into perspective. In case of stent II, a 1-cm stent with a 4 mm diameter, the stent has a total surface area of 34 mm^2^ containing a single layer of cells. Balloon contact with the vessel (1 cm length, 4 mm diameter) alone will damage an area 4 times the area of the stent, and this damage will protrude several cell layers deep, thus resulting in the loss of many more cells.

In a clinical setting of angioplasty, the use of a stent can be predicted for the majority of patients, with the exception of emergency cases. This two-stage procedure including a cell culture step may therefore not be limiting in most cases. Appropriate imaging (duplex ultrasound or CT angiography) can be performed in order to assess proper sizing. Stent dimensions will determine the number of cells required for confluent cell coverage. Growth characteristics of the cells can be monitored over time, thus allowing for appropriate planning of the clinical procedure. With the design of a stent packaging that allows infusion of a sterile cell suspension within a sterile packaging barrier, the need for large-scale bioreactors could be eliminated. The major limiting factor for clinical use is the time required for cell expansion [24]. Improving seeding efficiency and thus a reduction in the number of cells required for seeding will further speed up the entire procedure, resulting in prompter and more valuable treatment of the patient.

While both nitinol and 316L SS stents were studied, only the SS stent was afforded with grooves. It is technically possible to apply the same grooves to nitinol wires, yet at this stage the shape of the groove is still under development. Also in terms of deployment, SS stents are compatible with any angioplasty balloon, whereas deployment of nitinol stents requires dedicated delivery devices, making the grooved SS stent a good initial study subject. A major benefit of stents is the possibility of adjusting the stent design and stent strut topography to our needs. Stents also offer the option of bioabsorbability [19, 29]. Intimal hyperplasia after stenting is not only driven by EC and media damage, but also by flow disturbances due to the presence of the stent mesh [30]. Bioabsorbable stents would provide the advantages of current stents in terms of initial mechanical support, while omitting the disadvantage of permanently present artificial biomaterials. Use of fully bioabsorbable metals is as yet not common practice although studies to achieve controlled mechanical degradation with minimal cytotoxic effects are well underway [31–33].

## Conclusion

In an in vitro setting, retention of endothelial cells after seeding and after exposure to flow was significantly improved on a new grooved stent strut compared to smooth stent struts. Cell detachment due to stent deployment was similar for stent struts, regardless of stent strut geometry or deployment method. Cells that persisted after deployment were immediately flow resistant.

## Electronic supplementary material

Below is the link to the electronic supplementary material.

Supplementary material 1 (DOCX 11 kb)
