# Supplementary material for: Continuously Grooved Stent Struts for Enhanced Endothelial Cell Seeding
Source: Cardiovasc Intervent Radiol. 2017 May 3;40(8):1237–45. doi: 10.1007/s00270-017-1659-4 (PMC5489614; doi:10.1007/s00270-017-1659-4)
Supplement: Supplementary file 1 — Supplementary material 1 (DOCX 11 kb) [file 270_2017_1659_MOESM1_ESM.docx]

**Immunocytochemistry protocol**

Cells were grown to confluency and seeded onto gelatin (2% w/v) coated metal at 6x10^6^ cells/cm^2^ metal. After 16-24 h, seeded cells were fixed with ice-cold 80% acetone in water for 10 min. To block non-specific binding, 10% normal donkey serum (Abcam, Cambridge, UK) in PBS was added 30 min prior to staining. After washing in PBS, specimens were incubated overnight at 4°C with antibodies to von Willebrand Factor (vWF) (rabbit anti-human IgG, clone EPSISR15, 1:500 v/v, Abcam) and CD31 (mouse anti-human IgG1, 5 µg/ml, R&D Systems, Minneapolis, MN, USA), both diluted in PBS with 1% bovine serum albumin (BSA) from Sigma. Specimens were washed three times in PBS (1% BSA). Secondary antibodies goat anti-mouse (IgG H&L, Alexa Fluor 488 1:1000 v/v, Invitrogen, Carlsbad, CA, USA) and donkey anti-rabbit (IgG H&L, Alexa Fluor 488 1:1000 v/v, Abcam, both diluted in PBS with 1% BSA) were added for 1 h in the dark at room temperature. Cells on glass were washed and mounted onto glass slides using Fluoromount^™^ Aqueous Mounting Medium with DAPI (Sigma). Cells on stents were washed and submerged in PBS in 24-wells plates, a drop of Fluoromount with DAPI was added to the wells prior to imaging. Imaging was performed using a Leica DMI6000B inverted microscope. For assessment of background fluorescence and negative controls, the primary antibodies were omitted which resulted in absence of fluorescent signal. Images were obtained by stacking 10 to 20 images using ImageJ (extended depth of focus plug-in).
